# A Special Phase Detector for Magnetic Inductive Measurement of Cerebral Hemorrhage

**DOI:** 10.1371/journal.pone.0097179

**Published:** 2014-05-09

**Authors:** Gui Jin, Jian Sun, Mingxin Qin, Chao Wang, Wanyou Guo, Qingguang Yan, Bin Peng, Wencai Pan

**Affiliations:** 1 College of Biomedical Engineering, Third Military Medical University, Chongqing, China; 2 College of Electronic Engineering, Xidian University, Xi’an, China; University of California at Berkeley, United States of America

## Abstract

Cerebral hemorrhage is an important clinical problem that is often monitored and studied with expensive techniques, such as computed tomography (CT), magnetic resonance imaging (MRI), and positron emission tomography (PET). These devices are not readily available in economically underdeveloped regions of the world and in emergency departments and emergency zones. The magnetic inductive method is an emerging technology that may become a new tool to detect cerebral hemorrhage. In this study, a special phase detector (PD) was developed and used for cerebral hemorrhage detection with the magnetic inductive method. The performance indicated that the PD can achieve phase noise as low as 6 m° and a 4-hour phase drift as low as 30 m° at 21.4 MHz. The noise and drift decreased as the frequency decreased. The performance at 10.7 MHz was slightly better than that of other recently developed phase detection systems. To test the practicality of the system, the PD was used to detect the volume change in a self-made physical model of the brain. The measured phase shift was approximately proportional to the volume change of physiological saline inside the model. The change of the phase shift increased as the volume change and frequency increased. The results are in agreement with those from previous reports. To verify the feasibility of *in vivo* detection, an autologous blood injection model was established in rabbit brain. The results from the injection group showed a similar trend of increasing phase shift change with increasing injection volume. The average phase shift change induced by a 3-ml injection of blood was 0.502°±0.119°, which was much larger than that of the control group. The measurement system can distinguish a minimal cerebral hemorrhage volume of approximately 0.5 ml. All of the results demonstrated that the PD used with this method can detect cerebral hemorrhage.

## Introduction

Cerebral hemorrhagic stroke has high incidence, high morbidity and high mortality. This disease threatens human health and imposes severe economic burdens on the government and on families [1]. Currently, the noninvasive medical imaging modalities to detect the occurrence of cerebral hemorrhage include MRI and PET. However, MRI and PET are expensive, require large instruments and are available to only a small part of the prospective patient population in danger of developing hemorrhage [2], [3]. Magnetic induction tomography (MIT) is a new technique that may be used as a low-cost tool for non-invasive detection of cerebral hemorrhage [4]. The magnetic inductive phase shift method, which is one-dimensional MIT, is also widely studied. In magnetic inductive measurement, a signal source is used to output a certain frequency current into a transmitting coil to induce eddy currents in the measured object. The eddy currents will, in turn, produce a new magnetic field known as the perturbation field. These two fields, constituting a superimposed field, are sensed by a receiving coil, and the phase shift between the exciting field and the superimposed field is proportional to the conductivity of the object and the signal frequency. This phase shift, also called magnetic inductive phase shift (MIPS), is usually measured by phase measurement hardware [4].

The development of MIT and MIPS methods for biomedical use has much difficulties because the conductivities of biological tissues are notably low (σ<3 Sm^−1^); thus, the induced magnetic field is typically only 1% of the primary magnetic field at a frequency of 10 MHz [5]. When the frequency of the exciting signal is 10 MHz, the whole measurement system requires a phase measurement accuracy of at least 0.01° [6]. Highly accurate MIPS measurement hardware is necessary for an MIT or MIPS system.

Currently, various phase measurement methods are used in MIT or MIPS systems. The first type, consisting of commercially purchased off-the-shelf lock-in amplifiers, is widely used [7]–[9]. These amplifiers are very expensive, and their precision is not good at high frequency. The second type consists of off-the-shelf NI PXI systems. The ADC cards in the slots of the PXI platform are controlled by a LabVIEW program for signal acquisition. The phase difference is also calculated by the algorithms in the LabVIEW program. The new Graz MK2 system [10], [11], the Glamorgan system [12] and the Bath medical system [13] are based on the PXI platform. The costs of the PXI systems are also very high. The last method involves self-made FPGA-based systems. Because FPGA chips are good at high-speed data collection and signal processing, they are favored by many research teams. Patz and Watson [14] developed an FPGA-based direct digitizing signal measurement module for MIT. By adopting the LabVIEW quadrature demodulator, the phase noise of the system can reach 7.5 m°, whereas its phase drift is no more than 120 m° within 6 hours. In Trakic’s rotational MIT system [15], the data collection and phase calculation were completed in an FPGA development board. Wuliang Yin et al [16], [17] used an FPGA-based digital phase measurement method in all of their studies and obtained favorable results.

Our team has used the MIPS method to detect cerebral hemorrhage for many years and has developed a special PD for this purpose. The goals of the present study are to test the feasibility of using this PD and the MIPS method to detect cerebral hemorrhage and to study the relationship between the MIPS and the injection volume. The performance of the PD was measured and compared with the recently developed MIT systems. Experiments were performed to detect cerebral hemorrhage using a physical model and autologous blood injection in rabbit brain. The results are provided later in this study.

## Methods and Materials

### 1 Design of the PD

According to the dielectric dispersions of biological tissue and the frequency of the crystal filter, we selected three frequency points within the β-dispersion frequency range that are commonly used for studying biological tissue with the magnetic inductive method, including 1, 10.7, and 21.4 MHz [18]. The PD is especially designed to work separately at these three frequencies.

**Figure 1:**
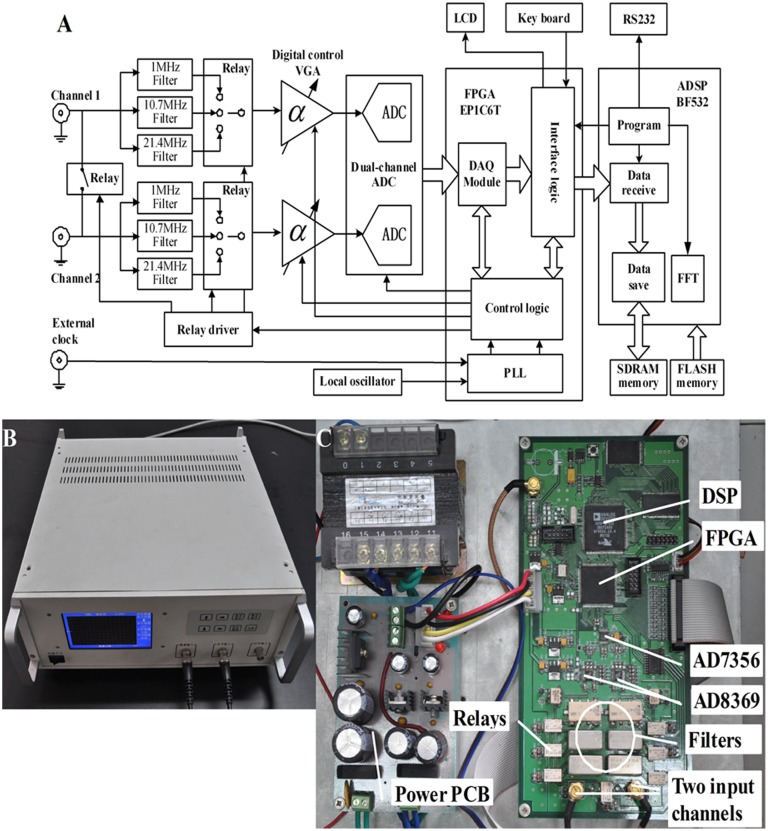
Schematic diagram (A), photograph (B), labeled internal structure (C), of the PD.

The schematic diagram is shown in Figure 1A. Two input signals first pass through different filters according to their frequencies and then are input into two digitally controlled variable gain amplifiers (VGA). The 1 MHz signal passes through an SCLF-4.7 low-pass filter (Mini-Circuits), and the signals at 10.7 MHz and 21.4 MHz pass through two PBP-10.7 and PBP-21.4 band-pass filters (Mini-Circuits), respectively. The relay controls the switch between the filters. Two AD8369s (Analog Devices) are used as VGAs. The AD8369 is a high-performance digitally controlled VGA for use at low frequencies to a –3 dB frequency of 600 MHz at all gain codes. The AD8369 provides a gain adjustable range of −10–35 dB in 3-dB increments when its load is 200 ohms. The filter and the amplifying circuits are the same for both channels. After being amplified, the two signals are converted by an AD7356 (Analog Devices), which includes two 12-bit AD converters and one high-speed serial peripheral interface (SPI). The dual-channel signals are sampled synchronously under the clock sequences provided by the FPGA. The converted data are first sent to the FPGA and then to the external SDRM through the DMA channels of the two dual-channel synchronous serial ports (SPORT0 and SPORT1) of the DSP. The DSP program first calculates the phase of each channel using fast Fourier transform (FFT) and then obtains the phase difference between the two channels. A Cyclone EP1C6T144 FPGA chip produced by the ALTERA Company is used. The FPGA also provides the clock and time sequences for the other circuits and interfaces. The DSP controls the operation of the entire system. The DSP switches the frequency, sets the gain and receives key values through the FPGA. When the keyboard is triggered, an interrupt signal is generated and sent to the DSP. In the interrupt service routine, 8-bit key values are read by the DSP. The DSP chip is an ADSP-BF532 (Analog Devices Inc.), which is a high performance 16-bit fixed-point DSP, up to 400 MHz, with adequate peripheral interfaces for easy use. Before each valid acquisition, a calibration sampling is initiated, and the relay between two channels is opened. The two channels sample the same signal, and the phase difference calculated at this time is only decided by the hardware circuits of the two channels. This phase difference is subtracted from the subsequent measurement value.

Because the input signals are all single narrow-band signals, the down-sampling method is used to relieve the pressure from the hardware circuits. Given that the sampling clock for the AD7356 is 60 MHz, and 15 clocks are needed for one conversion, the system sampling frequency is 4 MHz. The spectra of the signals at 10.7 and 21.4 MHz are moved to 1.3 and 1.4 MHz, respectively. MATLAB simulations indicate that achieving a precision of 0.005° requires at least 200,000 sampling points for three frequencies when FFT is used. The 200,000 sampling points used for one measurement are larger than the one-dimensional DMA’s maximum capacity for one transmission. Therefore, the two-dimensional DMA channels of SPORT0 and SPORT1 are used.

The calculated data are displayed on the LCD in real time. Twelve hours of data can either be stored in external flash memory or transmitted to a PC through the RS232 serial port. As shown in Figure 1B and 1C, all circuit boards are fixed in a metal shielded box (350 mm×320 mm×150 mm).

### 2 PD Phase Noise Measurement

Two output channels of an arbitrary signal generator AFG3252 (Tektronix, Inc.), were connected to the input channels of the PD. The AFG3252 is a dual-channel arbitrary signal generator with a phase precision of 0.01°. The phase noise was measured with the amplitude of one of the channels of the AFG3252 adjusted to between 50 and 500 mVPP. The second channel was kept constant at 500 mVPP. The phase difference of the two channels of the AFG3252 was set as 0°. The phase noise was defined as the average standard deviation of ten successive groups; each group included 20 measurements (∼2 min) and each measurement was not averaged.

### 3 PD Phase Drift Measurement

The setup of the measurement was the same as for phase noise measurement. The input voltage levels for both channels of the PD were 50 mVPP. The phase difference of the two channels was set as 0°. The phase drift was defined as the maximum change within 4 hours.

### 4 PD Phase Linearity Measurement

The setup of the measurement was the same as for phase noise measurement. To test the phase linearity, the phase of one channel of the AFG3252 was varied from 0° to 0.08° (by an increment of 0.01° each time). Ten measurements were averaged to determine the phase difference.

### 5 Cerebral Hemorrhage Detection Using the Physical Model

**Figure 2:**
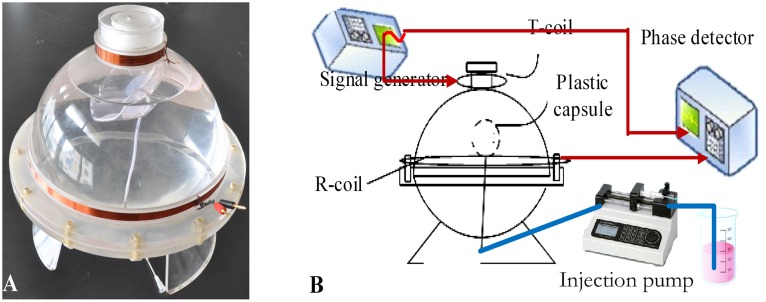
The physical cerebral hemorrhage model, (A). The experimental arrangement, (B).

The physical cerebral hemorrhage model is shown in Figure 2A. It consists of two different-sized coils with diameters D_1_ = 68 mm and D_2_ = 220 mm, coaxially centered at a distance d = 100 mm. The coil at the top of the model is a transmitting coil (T-coil), and a receiving coil (R-coil) is in the middle. The model is made of a soft plastic capsule centered inside of an organic glass sphere. The capsule, which can be filled with a maximum of 100 ml of solution, is connected to a conduit at the bottom of the glass ball, and the conduit is connected to a syringe pump. Solutions of varying volumes and conductivities can be injected into the capsule to simulate cerebral hemorrhage. The remaining volume of the model is partially filled with physiological saline (conductivity of 0.3 Sm^−1^) to simulate brain tissue. The volume of the model is similar to the volume of an adult’s head.

The experimental arrangement is shown in Figure 2B. One output channel of the AFG3252 was connected to the T-coil with amplitude of 5 VPP (a constant voltage source). The output of the R-coil and the other output channel of the AFG3252 with an amplitude of 1 VPP were connected to the two channels of the PD. Before measurement, 1500 ml of physiological saline (conductivity of 0.30 Sm^−1^) was injected into the model outside the capsule to simulate brain tissue.

Before the simulated hemorrhage experiment, we first measured the SINAD (Signal to Noise and Distortion) of the signal from the R-coil. The signal of the R-coil was input to one channel of a DAQ card PCI5124 (NI). A built-in function of SINAD in LabVIEW software was used to calculate the SINAD. The voltage input to the T-coil was regulated to make the signal voltage of the R-coil approximately 100 mVPP for all three frequencies. The SINAD measurements were repeated for three frequencies.

We then measured the phase drift. When the solution was static, the MIPS was continuously measured under the same conditions for 4 hours. The drift measurements were repeated for three frequencies. Each phase drift was defined as the maximum change of the MIPS within 4 hours.

The experimental procedure for hemorrhage detection was as follows. First, the system was allowed to equilibrate over 10 min, and then, 80 ml of physiological saline (conductivity of 1.10 Sm^−1^ to simulate the hemorrhage) was injected into the capsule at a constant rate of 1000 ml·h^−1^ and stopped for 10 min after the injection. Then, the injected saline was pumped out at the same rate of 1000 ml·h^−1^ and stopped for 10 min after the pumping. During the experiment, the MIPS was measured continuously. The experiment was repeated at 1, 10.7 and 21.4 MHz.

### 6 Ethics Statement of Cerebral Hemorrhage Detection in Rabbits

The Animal Experiments and Ethics Committee of Third Military Medical University approved all experimental protocols, and the care of the animals was carried out in accordance with the Declaration of Helsinki and IASP guidelines [19], [20].

### 7 Experimental Animals

Eight specific-pathogen-free (SPF) male New Zealand white rabbits, weighing 2.5±0.5 kg and aged 4–5 months, were purchased and randomly selected from the Animal Center of DaPing Hospital, Chongqing, China, and received humane care from a properly trained professional in the hospital. The eight rabbits were randomly divided into two groups: an injection group (n = 6) and a control group (n = 2).

### 8 Experimental Procedures

**Figure 3:**
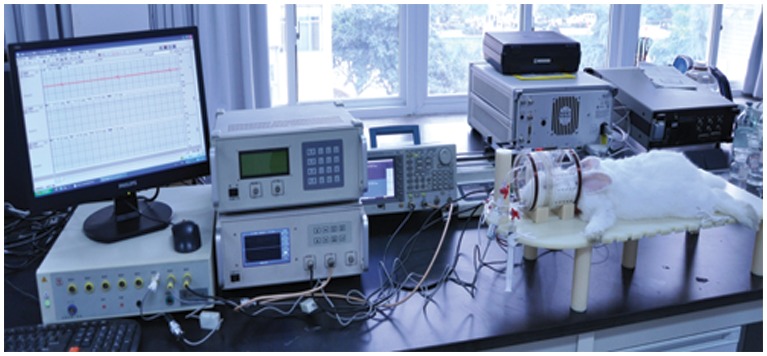
The experimental arrangement for cerebral hemorrhage detection in rabbits.

To detect hemorrhages in rabbit brain, a coil headgear was designed (see Fig. 3). The T-coil and R-coil (both, 10 winds, diameter = 100 mm) were coaxially placed and wound on a circular plastic former. The distance between the coils is 110 mm. The T-coil is placed close to the neck of the rabbit, and the R-coil is placed in the other end. To avoid inductive pickup induced by the aperture between the signal wires, the leads close to the joint of the coils were twisted.

The rabbits were first anesthetized via an injection of urethane (25%, 5 ml/kg) in the ear vein. A longitudinal incision was made on the median of the head, and the anterior fontanelle and coronal suture were exposed. A hole (d = 1 mm) was drilled 1 mm in front of the coronal suture and 6 mm from the midline. A total of 5 ml of fresh autologous blood was extracted from the subcutaneous vein of the hindlimb using a heparinized syringe. A needle (d = 0.7 mm) was introduced to an appropriate depth (H = 13 mm).

After the procedure, the rabbit’s head was placed in the center of the coil headgear. In all experiments, the coil headgear was positioned in such a way that the rabbit’s head was centered between the two coils. Then, the two coils were connected to the measurement system, which was the same setup as in the physical model detection. The excitation signal was 5 VPP, and a frequency of 10.7 MHz was selected. An RM6280C biological signal collecting and processing system (Chengdu Instrument Factory, China) was used to continuously measure the changes in the electrocardiogram (ECG) and heart rate variability (HRV). The RM6280C was controlled by a PC. The entire experimental arrangement is shown in Figure 3. All devices were started thirty minutes before the surgical procedure. When the experimental setup was ready, a syringe pump was used to continuously inject 3 ml of autologous blood into the injection group at a steady rate of 1 ml/3 min. The MIPS was measured throughout the experimental process. The ECG and HRV were also continuously monitored throughout the experimental process. The surgical procedure for the control group was the same as for the experimental group, but no blood was injected. For both groups, the baseline data were collected after the surgical procedure and before the injection (t = 0). The geometrical position between the rabbit’s head and the headgear was carefully maintained to be as similar as possible for all animals. The MIPS were recorded with respect to the baseline data. The fundamental hypothesis in this study is that the change in the MIPS will be representative of the average conductivity change in the brain with the increase in the volume of injected blood.

## Results

### 1 PD Phase Noise vs. Signal Voltage Level

**Figure 4:**
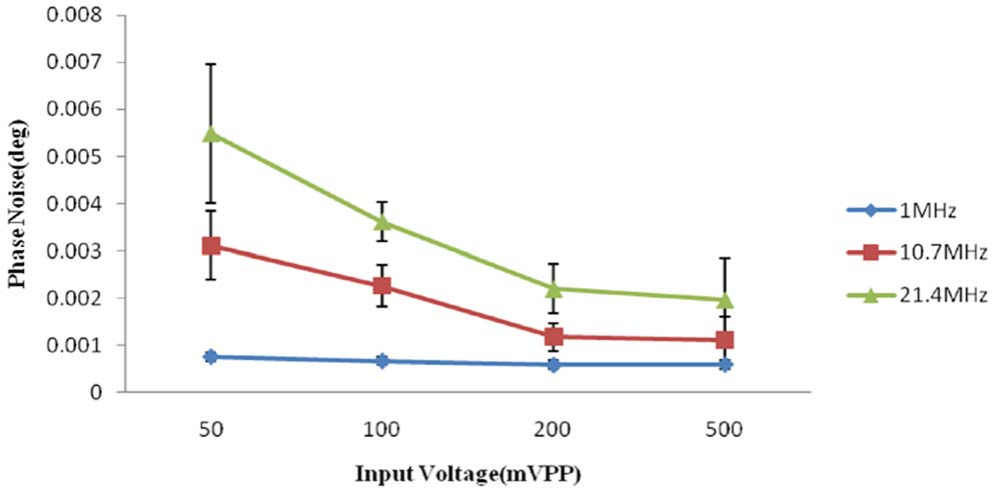
The measured phase noise versus the input voltage level.

The phase noise was measured against the input voltage level. The result is shown in Figure 4. The minimum phase noise was 0.585 m°±0.099 m° at 1 MHz, and the maximum phase noise was 5.480 m°±1.477 m° at 21.4 MHz.

### 2 PD Phase Drift

The phase drifts within 4 hours were approximately 9, 16 and 30 m° at 1, 10.7 and 21.4 MHz, respectively.

### 3 PD Phase Linearity

**Figure 5:**
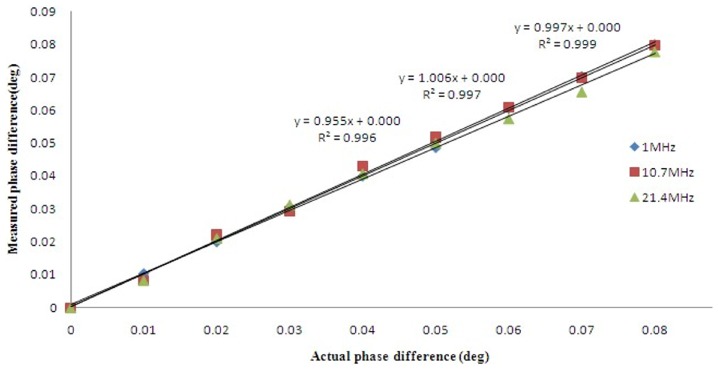
Phase linearity relationship between the set and measured phase difference.

The linearity performance is shown in Figure 5. A linear regression fit was applied between the set and measured phase differences. The R^2^ values were 0.9990, 0.9970, and 0.9960 for 1, 10.7, and 21.4 MHz, respectively.

### 4 Cerebral Hemorrhage Detection in the Physical Model

The SINADs of the signal from the R-coil were 37.9±0.067 dB, 34.6±0.23 dB, and 28.9±0.27 dB at frequencies of 1 MHz, 10.7 MHz and 21.4 MHz, respectively. Although the SINAD was very low for the three frequencies, the signal-to-noise ratio was improved after the signal was filtered in the PD.

**Figure 6:**
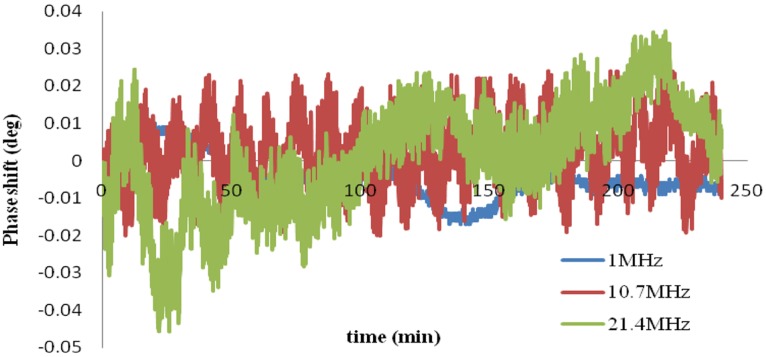
Phase drifts of the physical hemorrhage model over 4 hours without injection and pumping.

Figure 6 shows the phase shifts for the physical model over 4 hours at the three frequencies. The data were normalized to the average value. The phase drifts were 27 m°, 44 m° and 87 m° at frequencies of 1 MHz, 10.7 MHz and 21.4 MHz, respectively.

**Figure 7:**
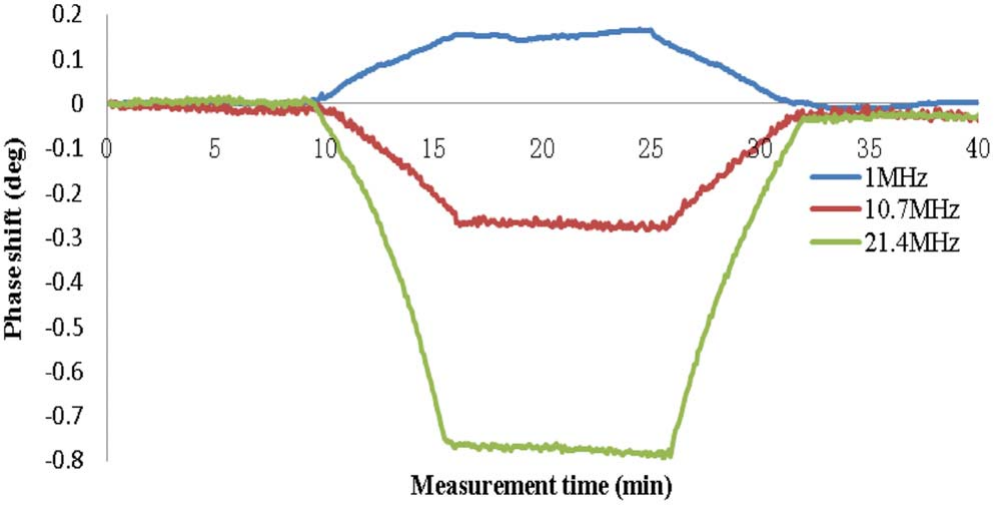
Phase shift caused by injecting and pumping 80 ml of solution.

Figure 7 shows the results of the hemorrhage detection. The data were normalized to the initial value. The changes in the MIPS caused by the injection of 80 ml of solution were 0.150°±0.0109°, 0.266°±0.0047° and 0.768°±0.0036° at 1, 10.7 and 21.4 MHz, respectively. Under the same conditions, the MIPS presented an approximately linear decrease as the volume of solution inside the capsule increased, whereas a decrease of the solution volume inside the capsule was accompanied by an approximately linear increase of the MIPS at frequencies of 10.7 and 21.4 MHz; however, the inverse was observed at 1 MHz. This may have occurred because the phase shifts at 10.7 and 21.4 MHz were opposite to those at 1 MHz. Although the best sensitivity was observed at 21.4 MHz, the worst phase drift occurred at 21.4 MHz. Therefore, we chose a frequency of 10.7 MHz for detection in the animal model.

### 5 Cerebral Hemorrhage Detection in Rabbits

**Figure 8:**
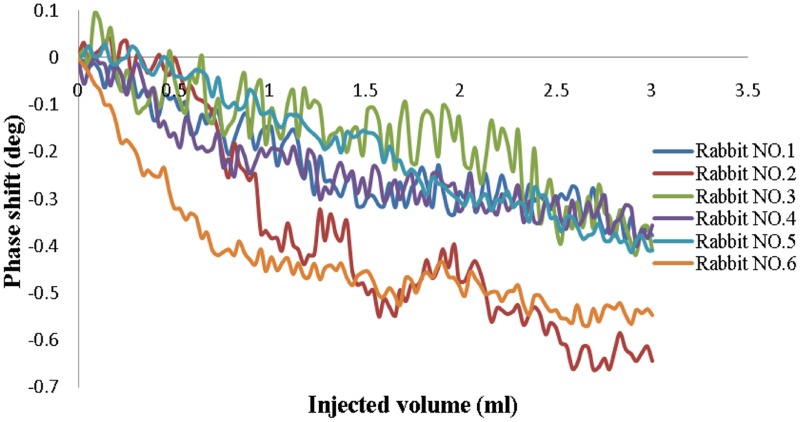
Phase shifts versus injected volume in the injection group.

**Figure 9:**
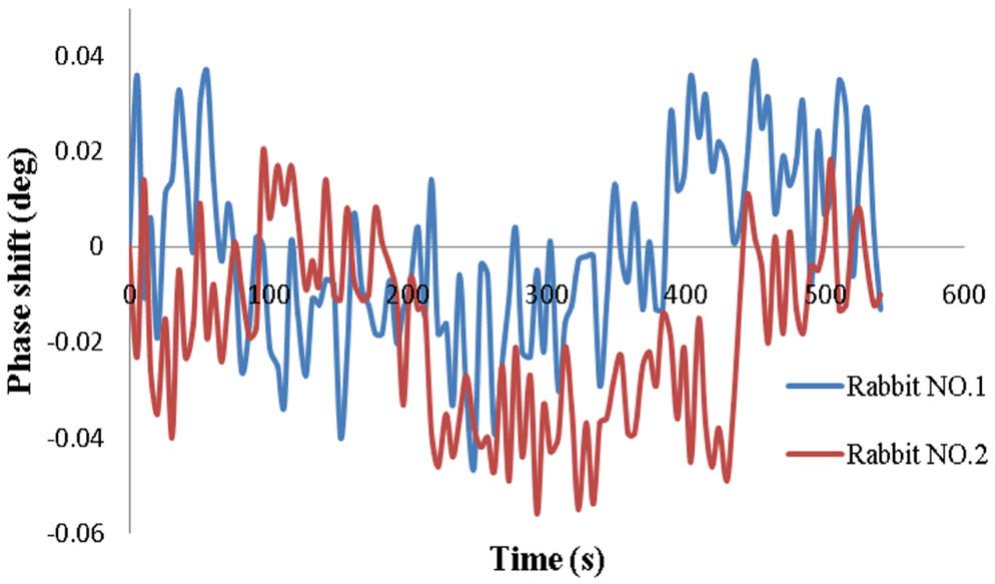
Phase shifts in two rabbits in the control group.

The results from the injection group are shown in Figure 8. The data were normalized to the baseline data and were not averaged. The HRV remained approximately level at 270 throughout the experimental process for all animals. Similar trends were found for all six animals, in that the MIPS decreased with increased injection volume. The changes in the MIPS with the 3-ml injection ranged from 0.387° to 0.702° (average 0.502°±0.119°). As shown in Figure 9, the phase drifts of the two rabbits in the control group over 9 min were 0.085° and 0.076° (average 0.081°±0.006°), which was much lower than the change in the injection group. Thus, we can conclude that the changes in the MIPS were indeed caused by the injection volume.

## Discussion

Figure 4 shows that the phase noise increases as the signal frequency increases and the input voltage level decreases. At 10.7 MHz, the noise is approximately 3 m° with an input of 50 mVPP, which is lower than the 4 m° noise performance of the Bath Medical System [13]. The maximum phase noise does not exceed 6 m°. The phase drifts within 4 hours are approximately 9, 16 and 30 m° at 1, 10.7 and 21.4 MHz, respectively. Wei et al [13] reported that at a frequency of 10 MHz, the maximum drift was 25 m° over 5 hours with a 50 mVPP input level for the Bath Medical System, 119 m° over 6 hours with a 65 mVrms input level for the Cardiff MK2 System, 102 m° over 30 min with 200 mA at the driving coil for the Philips Research System and 27 m° over 20 min with a −21 dBV input level for the Glamorgan System. Thus, the noise and drift performance of our PD is slightly better than those of other recently developed MIT systems. Figure 5 shows that the linearity performance is slightly worse than the R^2^ = 0.9998 reported by Patz et al [14] and the R^2^ = 0.9997 reported by Wei et al [13].

The results from the physical model show that the change in the MIPS increases as the volume in the plastic capsule increases. The higher the frequency is, the greater the change is. In 1999, the MIPS formula was derived by Griffith according to the electromagnetic model of a circular disk [21]. Consider the T-coil and R-coil positioned coaxially and separated by a distance 2*a*: a sinusoidal current, of angular frequency *ω*, flows in the T-coil, and the R-coil experiences a magnetic field *B*. Let a circular disk of dielectric of radius *R*, thickness *t*, conductivity *σ*, and relative permittivity ε_r_ be placed coaxially and centrally between two coils. The currents induced in the disk cause a perturbation, Δ*B*, in *B*. If the skin depth of the electromagnetic field in the dielectric is large compared with *t* (i.e., the interaction is weak), it can be represented as follows:
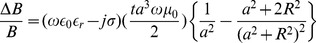
(1)where ε*_0_* and *µ_0_* are the permittivity and permeability of free space, respectively. Because ε_0_ is very low, the real part can be neglected. Given 
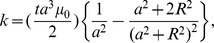
 so
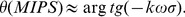
(2)When 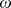 and 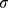 are kept still, MIPS is proportional to *k*. *k* increases with the increase of *t* and *R*; that is, *k* increases when the volume of the circular disk increases [21]. The results are consistent with formula (2).

The results are also consistent with those of Gonzalez et al. 2007 [22] and Flores et al. 2008 [23]. In Flores et al. 2008 [23], a similar physical head model was built to simulate hypoperfusion and bleeding in the brain, and inductive phase shift measurements were performed to detect changes of fluid volume in an internal glass sphere; these showed significant phase shift increases as a function of frequency and fluid volume in a frequency bandwidth of 1 kHz to 1 GHz. In Gonzalez et al. 2007 [22], the same results were found for five frequencies (40, 50, 100, 200 and 300 MHz).

For each curve at a given frequency, after injection and pumping, the data values all return essentially to baseline, indicating the system has high stability and repeatability. However, the phase noise of the MIPS is higher than that of the PD itself, most likely because of the sensitivity change induced by the movement of the internal capsule during injection and pumping, which occurs because the capsule is not fixed in the model [3].

The results of hemorrhage detection in rabbits (see Figure 8) show that the MIPS decreased with the injection of blood. The average change in the MIPS was 0.502°±0.119° with a 3-ml injection. For the control group, the average phase drift was 0.081°±0.006°. Thus, the measurement system can distinguish a minimal hemorrhage volume of 0.081°/0.502°×3 ml = 0.48 ml. However, it is clear in Figure 8 that the changes in the MIPS are different among the six rabbits and range from 0.387° to 0.702°, most likely because the position of the rabbit’s brain in the headgear could not be maintained exactly the same for all rabbits, which affected the sensitivity. Occasionally, incomplete anesthesia resulted in restlessness, hyperactivity and rapid breathing during the experiments. All of these factors led to high phase noise. In addition, the injection sites could not be fully consistent among all rabbits, which also affected the results. Moreover, the coils were not shielded in the experiments, and the results would be improved with the use of a shield screen. This experiment is preliminary; therefore, the postsurgical inflammation, cerebral blood flow and cerebrospinal fluid regulatory function were not considered. An induced cerebral hemorrhage model will be established in future experiments. Pathological and imaging studies will be conducted to further verify the practicality of this method. Nevertheless, this study clearly reveals the relationship between hemorrhage volume and the MIPS. The PD used with this method has the potential to detect cerebral hemorrhage.

## Conclusion

This study presents the development and performance of a special PD for cerebral hemorrhage detection based on the MIPS method. The PD achieves a better performance regarding phase noise and drift than the recently developed MIT systems. The results from hemorrhage detection in the physical model are consistent with previous reports. Cerebral hemorrhage detection in rabbits reveals that the measurement system can distinguish a minimal cerebral hemorrhage volume of approximately 0.5 ml, which is an exciting result. However, the animal experiment is preliminary, and postsurgical inflammation, brain hydration level, cerebral blood flow and cerebrospinal fluid regulatory function were not considered. An induced cerebral hemorrhage model in rabbits and pigs will be used in the future. A fixed setting for the animal’s head and a shielding screen should be added to the headgear. Because the sensitivity of the physical model detection is different from that of the animal model, no comparison can be made between the two. The sensitivity depends a great deal on the magnetic strength. The coils used in the two models are different. The diameter of the T-coil in the physical model is small; the magnetic field lines spread out and the magnetic strength is weak. Moreover, different combinations of T-coil and R-coil have different resonance frequencies. When the work frequency is close to the resonance frequency, the magnetic strength is increased. However, both model results reveal the same trend.

One limitation of the PD is its low speed. Because 200000 sampling points are used to perform the FFT calculation for one measurement in the DSP program, the time for a single measurement is approximately 3.0 to 5.0 s. An FPGA chip with a DSP IP core would be a good choice to reduce the measurement time. This type of chip will be used in our future work.
